# Promoting Mental Health Help-Seeking Behaviors by Mental Health Literacy Interventions in Secondary Education? Needs and Perspectives of Adolescents and Educational Professionals

**DOI:** 10.3390/ijerph191911889

**Published:** 2022-09-20

**Authors:** Lindy Beukema, Janne M. Tullius, Lies Korevaar, Jacomijn Hofstra, Sijmen A. Reijneveld, Andrea F. de Winter

**Affiliations:** 1 Department of Health Sciences, University Medical Center Groningen, University of Groningen, Antonius Deusinglaan 1/FA10, 9713 AV Groningen, The Netherlands; 2 Research and Innovation Centre for Rehabilitation, Hanze University of Applied Sciences, 9747 AA Groningen, The Netherlands

**Keywords:** mental health literacy intervention, adolescents, help-seeking, secondary education, educators, qualitative research

## Abstract

Mental health literacy (MHL) interventions in secondary schools may help to improve competencies that adolescents require to stay mentally healthy and seek help if mental health problems arise. These MHL interventions should be tailored to the needs of adolescents and educational professionals (EPs) to reach sustainable implementation and long-term effectiveness. However, evidence is lacking on these needs. Thus, our aim was to explore their experiences with, and perspectives on, mental health help seeking and needs regarding MHL interventions. We performed online focus group discussions and interviews with adolescents (*n* = 21; 13–19 years) and EPs (*n* = 12) and analyzed the data using directed content analysis. We identified three themes related to mental health help seeking: (1) Limited MHL competencies of adolescents, (2) Limited competencies of EP to provide mental health support, and (3) Limited mental health promotion in the school environment. We further identified three themes regarding MHL interventions: (1) Addressing basic mental health knowledge and skills, (2) Interactive and easily accessible, and (3) Sustainable implementation. Improving the MHL competencies of adolescents and EPs, and creating a mental health-literate school environment can promote adolescents’ mental health help seeking. Our findings highlight the importance of developing MHL interventions that are tailored to both adolescents’ and EPs needs.

## 1. Introduction

Mental health problems during adolescence can negatively influence school functioning, educational attainment, future labor opportunities, and social participation, leading to life-long disadvantages [1,2,3]. It is estimated that up to 50% of children and adolescents suffer from a mental disorder (at one point in their life) [2,4,5,6,7]. Despite this, adolescents are the least likely age-group to seek help for their problems [8,9,10]. Previous studies have identified several main barriers for adolescents in seeking help for mental health problems: (a) the incorrect recognition and identification of mental health problems and support, (b) stigma and embarrassment surrounding mental problems, and (c) a preference for self-reliance opposed to the professional help offered by general practitioners or mental health services [11,12,13,14]. These barriers can hinder adolescents in adequately managing their mental health, inhibit the use of mental health services, and lead to untreated mental health problems [11,15,16].

Strengthening the mental health literacy (MHL) of adolescents may help to maintain mental health and, if needed, overcome help-seeking barriers [11,15]. MHL has been defined as “the range of cognitive and social skills and capacities that support mental health promotion” and includes the ability to understand how to obtain and maintain positive mental health; understand mental disorders and their treatments; decrease stigma related to mental disorders; and enhance help-seeking efficacy (knowing when and where to seek help) ([17], p. 155). Strengthening MHL in adolescents can thus be an effective strategy to overcome barriers to help-seeking, manage mental health adequately, improve help-seeking efficacy, and intervene promptly in worsening mental health [18,19,20,21].

The school may be an optimal setting for improving adolescent MHL and in recent years, more and more evidence-based MHL interventions have been implemented here. Despite this being a positive development, in many countries, including the Netherlands, no school-based MHL intervention is available. The exceptions are programs focusing on reducing stigma and/or specific disorders, e.g., depression [22]. Furthermore, the current MHL interventions in the school setting vary in implementation success and (long-term) effectiveness [23,24]. For the successful development and implementation of new and existing MHL interventions in secondary education, it is essential to tailor MHL interventions to the needs of adolescents and educational professionals (e.g., educators, school support staff) [25,26,27]. To do so, first, the experiences with and perspectives of mental health help-seeking behaviors and needs for MHL interventions of adolescents and educational professionals (EP) must be assessed. However, very little qualitative research has been performed exploring these perspectives in one study, as previous research has mainly focused on examining adolescents’ and educational professional’s perspectives in a quantitative manner, separately for the two groups, and/or in primary school settings [21,28,29]. Such research can highly facilitate the effectiveness and longevity of new and existing MHL interventions and thus create effective strategies to overcome the help-seeking barriers among adolescents, normalize the conversation of mental health in education, and improve access and quality of help-resources [25,30,31,32].

Therefore, the aim of this study was to explore mental health help-seeking experiences and perspectives and the needs regarding MHL interventions from both adolescents and educational professionals to inform the development and implementation of MHL interventions.

## 2. Materials and Methods

### 2.1. Study Design

For this study, we used a qualitative approach to assess the mental health help-seeking experiences and perspectives and the needs regarding MHL interventions in secondary education of adolescents and educational professionals (EP) through online focus group discussions and interviews (from here on referred to as OFG). We performed the design, analysis, and reporting according to the COREQ checklist [33]. The study was carried out in accordance with the Helsinki Declaration and ethical approval by the Medical Ethics Review Board (UMC Groningen, Groningen, The Netherlands) was obtained prior to the study (reference number: M17.213729 and 202000154). Informed consent was obtained from all of the participants (or their caregivers, if younger than 16).

### 2.2. Participants

The data were collected during the COVID-19 pandemic in the Netherlands (March 2020–June 2021). The participants were recruited through various social media channels (e.g., Facebook, Instagram) and snowballing techniques. Inclusion criteria for the adolescent participants included being between 13 and 19 years of age (i.e., the age range of adolescents attending secondary school in The Netherlands) and attending secondary school. Inclusion criteria for the EPs included working in secondary education in the Netherlands. All of the adolescent participants were rewarded with a EUR5 gift card. The researchers had no relationship with the participants.

Twenty-one adolescents participated in the OFG. Initially, 38 adolescents signed up for the OFG, however 17 adolescents (44.7%) did not respond to the invitation of the OFG. Twelve EPs participated in the OFG. A total of 28 EPs signed up for the OFG of which 57% did not respond anymore or changed their mind about participation. The high non-response rates are probably due the high workload of educational professionals and the measures to contain the COVID-19 pandemic in the period in which we performed this study (e.g., online contact and thereby lack of personal connection and of school rhythm).

### 2.3. Study Procedure and Measures

#### 2.3.1. Adolescents

The OFG with adolescents were held online on the platform Collabito (https://app.collabito.com/; accessed on 1 March 2020), using text-based live chats without audio or video to ensure the participants’ anonymity. Before the OFG, the participants gave consent to participate (if under the age of 16, their guardians gave consent) and were asked to fill in a questionnaire regarding their background information via the tool UniPark. The background information included the participants’ age, educational level, school grade, ethnicity, zip code, and self-reported current mental health. The educational level was categorized as lower vocational and lower secondary (VMBO), intermediate secondary (HAVO), and higher secondary education (VWO). For more detail on the Dutch educational system, see Veldman et al. [34]. For self-reported mental health, the participants were asked to rank their level of somberness (‘In the last three weeks, have you felt somber?’), worry (‘In the last three weeks, have you felt worried?’), and interest (‘In the last three weeks, have you felt like you do not have any interest anymore in the things you are usually interested in?’) on a 10-point Likert scale from 0 (‘All the time’) to 10 (‘Not at all’). The mental health questionnaire was based on the MINI-Scan, a validated, semi-structured psychiatric diagnostic assessment instrument [35].

The OFG were semi-structured, guided by a protocol and led by the first authors (LB, female; JMT, female). They were structured into two discussion rounds including open and closed questions. First, participants were asked about their mental health and mental health help-seeking experiences in and outside of school. Second, they were asked about their needs regarding MHL interventions. The adolescents were encouraged by the researcher to respond to each other and discuss their opinions. We held four OFG with two to four adolescents and nine online interviews (due to no-shows). The duration of the OFG varied between 50–120 min, depending on the group size and the extent of the participants’ answers. The participants were repeatedly offered breaks throughout the duration of the focus groups to preserve the levels of focus on the task at hand. Data collection continued until data saturation was reached. We considered data saturation to be reached when a new OFG did not yield new information on top of the already obtained information [36].

#### 2.3.2. Educational Professionals

The OFG with EPs took place in an online environment (Microsoft Teams). Prior to the OFG, the participants were asked to fill in a survey via the tool UniPark to give consent to participate and provide information on their demographics (e.g., role in education, school level, previous mental health training). The OFG was semi-structured, led by the first authors (LB, female; JMT, female) and guided by a protocol including open-ended questions regarding three main topics: (1) Mental health and help seeking at school, (2) Current mental health promotion in secondary education, and (3) Suggestions for improving introduction of a MHL intervention for adolescents and EPs.

The OFG began by presenting the participants with quotes stemming from the OFG with adolescents. Second, the participants were asked to share their experiences with mental health problems at school, what they perceived as the main barriers and facilitators for adolescents to seek help and for EPs to provide support, and what ideas they had to reduce these barriers. Finally, the participants were presented with an existing intervention aimed at improving MHL of adolescents and EPs (The Guide, [20]) and were asked to reflect on the content and what would be necessary for sustainable implementation. PowerPoint slides were used to guide the discussion in a semi-structured way. Five OFG were held with two to four EPs each. The duration of the OFG varied between 50–120 min, depending on the group size and the extent of the participants’ answers. A break of 10 min was held after the first half of the OFG to ensure levels of focus of the participants. Data collection continued until data saturation was reached. Data saturation was considered to be reached when new data collected did not yield any new information [36].

### 2.4. Data Analysis

The analysis of the transcripts of the OFG with adolescents and EPs was completed separately, but the same procedure was used. The analyses followed the principles of directed content analysis, based on previously established gaps in the literature, and were completed by the first authors, LB and JMT, using Atlas.ti computer software (version 9, ATLAS.ti Scientific Software Development GmbH, Berlin, Germany) [37]. First, both LB and JMT independently read all of the transcriptions to examine key topic patterns and relationships. Based on this, a draft codebook with distinctive codes and code groups was set up. This draft codebook was then used to code transcripts of two OFG and to compare the codes. The data that could not be coded were identified and either labeled as a new code or added as a subcategory of an existing code. The draft codebook was refined until consent among the authors was reached. The rest of the transcripts were then coded by either LB or JMT, after which the codebook was refined once again. During the analysis, both LB and JMT formulated candidate themes separately based on the codebook. LB and JMT discussed their identified candidate themes to review, refine, and define the main themes. Finally, the proposed main themes were discussed with the rest of the authors until consensus was reached on the final main themes.

## 3. Results

### 3.1. Sample Characteristics

The adolescent sample consisted of 21 adolescents with a mean age of 16.1 years (standard deviation, SD: 1.9). Most of the sample identified as female (76.2%) and were of Dutch ethnicity (81.0%). Of the sample, 52.4% attended the highest secondary school level (VWO in Dutch), 23.8% attended intermediate secondary education (HAVO) and 19.0% attended the lower vocational education level (VMBO). The mean self-rated mental health at the time of participation was 5.8 (SD 3.5) for somberness, 6.1 (SD 3.1) for worry, and 5.7 (SD 2.5) for interest, showing a great variation within the sample.

The educational professional sample consisted of 12 EPs from the Netherlands of which six were educators and six were school mental health counselors or coordinators (i.e., coordinators of the mental health counselors in their school). Most of the participants identified as female (75%). The participants worked in schools of various levels: Four in lower vocational education (VMBO) and eight indicated that they worked on all three educational levels (VMBO, HAVO, VWO).

### 3.2. I: Experiences with and Perspectives on Mental Health Help-Seeking in School

Table 1 provides the main themes accompanied by illustrative quotes. The analysis of the OFG with adolescents and EPs yielded the same three main themes in terms of mental health help-seeking experiences in secondary school and could therefore be summarized into: (1) Limited MHL competencies of adolescents, (2) Limited EPs competencies to provide mental health support, (3) Limited mental health promotion in the school environment.

#### 3.2.1. Theme 1: Limited MHL Competencies of Adolescents

The adolescent participants frequently mentioned that not being able to recognize mental health problems (e.g., depressive symptoms) and their causes, might be a barrier for their peers to seek help. They also had varying opinions about whether someone with depressive symptoms simply had “a bad week” or whether it indeed was someone with a depressive disorder. In particular, the participants without prior mental health problems more often referred to it as something temporary, such as a bad mood.

EPs also agreed that adolescents frequently lacked recognition of their own mental health problems and of the appropriate time to seek help. In addition, according to the EPs, mental health problems have been accelerated in recent years by the social pressure that adolescents experience nowadays to perform well, for example through social media, the home environment, or their peers.

#### 3.2.2. Theme 2: Limited Competencies of Educators to Provide Mental Health Support

The adolescent participants frequently mentioned that they thought EPs lacked training and competencies to handle situations that involved adolescents with mental health problems. Some adolescents also reported that the perceived limited competencies of EPs led to a lack of trust in them as a help resource. Only a few participants described having an educator who was a great source of support for their mental health issues, while others did not perceive any of their educators as an available source.

All of the EPs acknowledged that they did not feel competent to recognize mental health problems in adolescents, and to handle situations involving adolescents with mental health problems in the school context. Almost all of the participants agreed that there was insufficient training for EPs on mental health, both during their teacher training and as continuing education.

#### 3.2.3. Theme 3: Limited Mental Health Promotion in the School Environment

The adolescents reported that there was a lack of openness regarding mental health as it was rarely or never a topic of discussion in their school environment. The participants, especially those who had previous experience with mental health problems, spoke often about the importance and desire of “normalizing” and “opening the conversation” about mental health problems in school. The adolescent participants also often spoke about how they perceived the negative attitudes of others towards mental health. They related this to perceiving help seeking as shameful and as a barrier to speaking about their own problems, as well as a potential reason for others not to speak up or seek help.

For some of the participants, it was unclear where in school they could go to for help. Many mentioned that they were aware of a guidance counselor, but that they did not know who that person was or how to reach them. A few of the adolescent participants were aware of the care team that was in place at their school and what structures they could follow if they needed help.

The EPs acknowledged that due to time constraints and limited resources in schools, mental health or social-emotional development is currently not sufficiently addressed in the Dutch school system. They perceived that the priority and focus in secondary education lies on the transfer of knowledge, results of the final examinations, and the number of graduates of a school. Many furthermore reported that the current curriculum and educational system is “nailed shut” with few opportunities for societal and citizen skills or even social-emotional development. Consequently, according to the EPs, mental health becomes a topic that is not normalized and associated with shame among students and EPs. As a result, the EPs had the impression that there is a lack of trust of adolescents in EPs and other adults to disclose their mental health problems.

Some EPs reported that they recognized that the support structures were often unclear to adolescents. Many EPs also mentioned that they themselves were not aware of every aspect of the support available, for example that every school has an assigned youth health care physician. The EPs further raised the issue that their role in the mental health care process is often unclear to them. Some EPs saw it as their role to be a person of trust and a first-help resource to students, some noted that they felt it was part of their responsibility to signal mental health problems and refer students to appropriate help sources. For others, however, being an educator only entails the teaching of a subject (e.g., math, science, English) without having the task of signaling and referring students with mental health problems. In addition, the EPs frequently criticized the lack of referral protocols, unclear mental health care structures, and lack of low-threshold support services (“stepped care”). Cooperation and communication between the different parties such as EPs, the school administration, mental health care services, youth health care physician, community services, and parents were sometimes described as difficult and complex. Others experienced no issues.

### 3.3. II: Needs Regarding MHL Interventions in Secondary Education

The main themes that emerged from OFG with adolescents and EPs regarding their needs for MHL interventions led to similar themes and could therefore together be summarized into (1) Addressing basic mental health knowledge and skills, (2) Interactive and easily accessible interventions, and (3) Sustainable implementation. Table 2 provides the main themes accompanied by illustrative quotes.

#### 3.3.1. Theme 1: Addressing Basic Mental Health Knowledge and Skills

Most of the adolescent participants were in favor of learning more about mental health as they perceived it as helpful and an interesting and relevant topic. The participants mentioned that they wanted to learn about mental disorders, how to help others and themselves, and about the available resources in and outside of school. This was perceived as essential knowledge to positively change people’s attitudes towards talking about mental health and seeking help. In addition, it was frequently mentioned by adolescents that EPs should be included in a mental health literacy intervention. Furthermore, all of the participants agreed that EPs should receive training regarding mental health to educate them about the seriousness of mental health problems, and how to be more sensitive towards mental health problems in adolescents.

Most EPs agreed that there was a need for intervention approaches addressing the abilities to cope with or address mental health problems for both adolescents and EPs. Many ideas involved a multi-level approach: a school-based intervention aimed at improving both EPs’ and adolescents’ mental health skills and competencies. Basic knowledge about mental health and help seeking and, for example, being able to detect the difference between regular daily stressors and depression, was perceived as a useful tool to learn about mental health in the school environment.

#### 3.3.2. Theme 2: Interactive and Easily Accessible

A recurring theme among the adolescent participants was that a mental health intervention should be applied in an interactive, engaging, and light-mannered, accessible way with the right balance between enforcing the seriousness and avoiding the over-medicalizing of mental health. According to them, this could prevent negative attitudes from adolescents towards such an intervention. This was confirmed by the EPs who stated that it was most important to them that a MHL intervention at school would be low-threshold and easily accessible.

Many of the adolescent participants also agreed that an external individual, who is an expert by experience (with mental health problems) and has adequate knowledge on and training in the subject, should be involved.

#### 3.3.3. Theme 3: Sustainable Implementation

All of the adolescent participants agreed that a mental health intervention should be integrated in the curriculum of secondary school. Diverging opinions were expressed on the frequency of such lessons: Workshops once every couple of months, once a month, or even a fixed class about mental health, just like Math or English. There was consensus that an awareness campaign once a year is not effective.

EPs further indicated that sustainable and sufficient implementation at school were the most important characteristics of a successful MHL intervention aimed at EPs and adolescents. Many of the participants emphasized that a program should be cost-effective, easy to apply for EPs, and repeated frequently at different times throughout one’s academic career. Furthermore, some of the participants preferred having a full subject aimed at mental health literacy while others wished that this topic could be integrated naturally in all school subjects (e.g., Math, English, biology, drama, etc.). There was a consensus that mental health should not only be discussed once a year at a “Mental Health Day” or annual awareness day as this would add little.

## 4. Discussion

Our study examined adolescents’ and EPs’ perspectives of and experiences with help seeking for mental health problems and needs regarding mental health literacy interventions in secondary education. In regard to their perspectives and experiences, both EPs and adolescents perceived limited MHL competencies of adolescents. Second, both EPs and adolescents also perceived limited competencies of EPs to provide mental health support. In addition, adolescents and EP both experienced limited mental health promotion in the school environment. Regarding MHL interventions, first, adolescents and EPs reported a need for learning basic mental health knowledge and skills, second, they reported a need for interactive and easily accessible interventions, and third, a need for sustainable implementation.

### 4.1. Experiences with and Perspectives on Mental Health Help-Seeking in School

First, both the adolescents and EPs found that adolescents have limited MHL competencies, as they frequently have difficulties recognizing mental health problems and partaking in appropriate help-seeking behaviors. This was perceived as a barrier for adolescents to mental health help seeking in the school environment. This finding confirms previous research that the levels of mental health literacy of adolescents are often insufficient, however this has not previously been reported from the perspective of EPs [38,39,40]. Our finding, based on the perspectives of both adolescents and EPs, underlines the need to strengthen MHL in adolescents in the school setting as a potential effective strategy to promote mental health help-seeking behaviors.

Second, both the adolescents and EPs experienced that EPs lack competencies to support adolescents with mental health problems. This finding corresponds to previous findings that EPs rate their training in mental health as inadequate, even though they themselves perceive it as part of their educating role to address mental health problems in their students and view the topic of student mental health as serious [29,41,42,43]. However, these findings have not previously been confirmed from the perspective of the adolescents. Our findings indicate that the lack of these competencies in educators negatively affects adolescents’ trust in them as a resource for mental health support. Our study therefore highlights the pressing need to provide (additional) training on mental health to EPs to potentially overcome adolescents’ barriers to mental health help-seeking behaviors in secondary education.

Third, we identified that both the EPs and adolescents experienced limited mental health promotion in the school environment. We identified contextual factors, such as a lack of openness, unclear support sources, time constraints, and insufficient referral structures, that hindered the adequate promotion of mental health. On the one hand, these factors (e.g., unclear support sources and referral structures) lead to inadequate support for adolescents with mental health problems, while on the other hand, they can lead to shame and stigma (e.g., lack of openness and time constraints), and thus limit mental health help-seeking intentions in schools. Previous research identified individual (e.g., stigma and embarrassment, self-reliance, lack of MHL) and social factors (e.g., school, family, peers) that limit adolescents’ mental health help-seeking behaviors [11,30,44], to which our findings add more specific insights about the role of contextual factors within the school environment and the limited promotion of mental health.

### 4.2. Needs Regarding MHL Interventions in Secondary Education

Regarding MHL interventions, first, the adolescents and EPs both emphasized their need to learn basic mental health knowledge and skills by means of such interventions. This finding aligns with previous studies assessing only teachers’ perspectives, while our study shows that adolescents perceive this in a comparable way, indicating the pressing need for MHL interventions in the school setting [28,45,46]. Second, both the adolescents and EPs indicated that a MHL intervention should be easily accessible, and applicable without being too heavy or serious. Our findings align with existing evidence that adolescents prefer low-impact and accessible resources, self-help tools, and strategies that positively influence stigmatizing beliefs [15,47]. Third, adolescents and EPs emphasized the importance of sustainable implementation of a future MHL intervention by means of integrating an MHL intervention in the regular school curriculum. This can support a more systematic change towards normalizing mental health within the school system [20].

Overall, this study highlights that adolescents and EPs have similar experiences and needs regarding potential MHL interventions in school, which serves as an important starting point for the development and implementation of new interventions or the adaptation of existing interventions. More specifically, this implies that new and existing interventions should ensure that the following aspects are taken into consideration: (1) Promote adolescents’ and EP’s MHL, (2) Promote a safe environment at school for mental health, and (3) Highlight basic knowledge and skills, an accessible and interactive format, and provide sustainable implementation.

#### 4.2.1. Strengths and Limitations

This study has several strengths. First, this qualitative study assessed both adolescents’ and EPs’ perspectives and experiences regarding mental health help-seeking behaviors in secondary education and needs for MHL interventions in a Western European country, namely the Netherlands. Therefore, our results can be applied to other contexts that share a similar cultural and educational system. Second, by using an anonymous online setting for the adolescents, we created a safe environment to express opinions, which limited the likelihood of socially desirable answers and thus promoted validity. In addition, the OFG setting gave the EP participants the opportunity to express their experiences in a way that they could not have done in a quantitative study design approach. Their lively interactions throughout the OFG provided us with a rich dataset, acquiring a complete picture of adolescents’ and EPs’ perspectives, experiences, and needs. Last, the deductive analysis allowed for an open exploration of our findings.

Some limitations of this study should also be considered. First, our recruitment strategy (e.g., social media and snowballing) may have led to a somewhat restricted sample as these strategies most likely yield participants with similar characteristics due to the participants being in the same social networks. In addition, this could have influenced the data saturation as participants with similar characteristics are more likely to give similar answers. Thus, even though we reached data saturation with our sample, a more balanced sample might have yielded other additional experiences and needs which may be explored with future research [48]. Second, the study was performed during the COVID-19 pandemic, which may have resulted in higher awareness and reflection of mental health problems, and a more negative perception of school support.

#### 4.2.2. Implications and Recommendations

The findings of our study offer insights into the perspectives and experiences of adolescents and EPs regarding mental health help seeking and their needs for MHL interventions in secondary school. First, our finding that adolescents and EPs have limited MHL competencies shows that mental health literacy interventions in secondary education should be aiming to support the mental health competencies of both adolescents and EPs. Strengthening their abilities might be an important first step to promote help seeking in the school environment and break the vicious cycle of mental health help seeking barriers in school. In addition, as mental health has a significant impact on the school functioning of adolescents, strengthening their MHL competencies has the potential to improve their school participation and future educational outcomes.

Second, our finding of limited mental health promotion in the school environment shows the current shortcomings regarding mental health in secondary education. As suggested by O’Reilly and colleagues, schools and EPs (both teaching and support staff) should be part of a multi-agency approach towards the promotion of adolescent mental health and well-being and play a key role in identifying signs and symptoms of youth mental health issues and referring them to the appropriate help resource [25,45]. Mental health promotion should become a priority through increasing the available resources at schools and clarifying EPs’ role in the mental health care process and referral procedures. More open communication about mental health, and rectification of false beliefs and negative stigma related to mental health (i.e., through MHL intervention) is equally recommended.

Third, our findings regarding MHL intervention needs (e.g., basic knowledge and skills, an accessible and interactive format, sustainable implementation) highlight the need for tailoring interventions towards the needs and perspectives of the end-users. Overall, our study shows the importance of including both adolescents and EPs in the research as well as the development and implementation process of an MHL intervention in secondary education. This combined perspective, by means of a co-creation process, can thus facilitate the sustainable implementation and long-term effectiveness of MHL interventions.

## 5. Conclusions

This study shows a need for improving the MHL competencies of adolescents as well as of EPs, and for creating a mental health-literate school environment to promote adolescents’ mental health help-seeking behaviors. Moreover, our findings highlight the importance of developing MHL interventions that are tailored to both adolescents’ and EPs’ needs. This can yield acceptable, accessible, and sustainable implementation and development of MHL interventions that are effective in the long-term.

## Figures and Tables

**Table 1:** Experiences with and perspectives on mental health help seeking in school.

Main Themes	Quotes Adolescents	Quotes Educational Professionals
Limited MHL competencies of adolescents	“Because they think their situation is not as bad as others’, and that they think they are overreacting.” (A3)	“I think that students themselves aren’t really aware of it, either. If students aren’t even aware themselves that they are about to sink into a depression.” (EP12, educator and mentor ^1^)
Limited competencies of educators to provide mental health support	“I wouldn’t recommend talking to someone at school. To talk about it, yes, but not at our school. They are just not very good with these things.” (A8)	“I wouldn’t say that I am very good at identifying certain problems. If it’s about ADHD, ADD and ASD, I can do that relatively easily. [..] But if we’re talking for example about anxiety disorders, depression or perhaps even eating disorders... [I am not capable to identify those].” (EP10, educator and mentor) “The average teacher is also not trained for this.” (EP3, coach/trainer)
Limited mental health promotion in the school environment	“There is probably someone at our school for this (next to your mentor), but even after seven years I honestly have no idea who that is.” (A3) “I noticed that myself, when my mentor ^1^ told the class that there was test anxiety training. The whole class started to mumble a little bit about how that was nonsense, et cetera and that they all didn’t need that. […] I finally signed myself up for the training but started doubting it because of all the comments from the class.” (A5) “Mental health problems and invisible physical problems are so badly accepted. People don’t understand that it’s not ‘just in your head’ when you have a depression. You also don’t say that asthma is ‘just in your lungs’.” (A4)	“At a certain point, as an educator and as mentor ^1^, you have to know your limits of what you can do. […] You are not a care provider in that sense. That is also not our expertise. So, I think that there is also a limit to where you can help.” (EP2, educator) “I think it’s also time pressure and workload. I think a lot of teachers already have so much to do and then wonder where they have to get time from [to pay attention to mental health issues of students]. People are afraid that it will come with a lot of extra work.” (EP5, educator) “The knowledge transfer is so important. The Ministry already asks a lot from schools; a lot is just required. And the culture, at least at our school, also focuses so much on studying for grades, showing progress. You can’t express the social-emotional [progress] in grades.” (EP4, educator)

^1^ In the Dutch school system, a mentor is a teacher who is assigned to a specific class, who keeps track of each student’s progress, has contact with parents, and provides support when necessary.

**Table 2:** Needs for MHL interventions in secondary education.

Main Themes	Quotes Adolescents	Quotes Educational Professionals
Addressing basic mental health knowledge and skills	“[Learning] about how you can help someone, how you can recognize that someone is dealing with something, and what kind of problems you can have.” (A14) “Not only students should be taught about mental health, also the teachers need to be informed better. So that they can also recognize the signs that may point to depression, anxiety or stress, etc.” (A5)	“Not even about disorders, but just simply about who you are and how you are feeling, because that’s where it often starts. And it should be structural; not just for six or eight weeks, no, the whole time they’re in school here with us.” (EP4, educator) “That it’s normal to be sad or stressed sometimes and learning how to cope with that.” (EP9, educator)
Interactive and easily accessible	“It should not become one big show. Mental health problems are serious, but by making it really dramatic the barrier will just be higher, and that could trigger negative experiences in some students. […] It should be really factual, I think. Or a type of class like philosophy. The subject matter is presented really factual, and afterwards the students that have interest in the topic can discuss it in the classroom.” (A4) “Maybe examples of personal stories of people who have had psychological problems. Personal stories are captivating to hear and can work inspiring.” (A1)	“Teachers like to be served bit-sized chunks with programs like this and like to serve those themselves. Or at least, I think that’s how it should be done.” (EP2, educator)
Sustainable implementation.	“I would say once every two months, but also monthly checkups on the students. And that there are also projects about this topic.” (A15) “[…] For example, during a class like civics class, or just during the mentor* hour. […] Every week is a bit excessive, but just some repetition occasionally and starting the conversation about it.” (A16)	“I would make it a regular course in school, a part of the regular education. It [mental health problems] is taking up more space in our job and it would be a good solution to also train teachers.” (EP8, support coordinator) “It should be a fixed aspect of the teacher education, and it should not be an afterthought.” (EP4, educator)

## Data Availability

The data presented in this study are available on request from the corresponding authors. The data are not publicly available as specified on the informed consent forms from study participants.
